# Spider Phobia: Neural Networks Informing Diagnosis and (Virtual/Augmented Reality-Based) Cognitive Behavioral Psychotherapy—A Narrative Review

**DOI:** 10.3389/fpsyt.2021.704174

**Published:** 2021-08-24

**Authors:** Jonas Hinze, Anne Röder, Nicole Menzie, Ulf Müller, Katharina Domschke, Matthias Riemenschneider, Michael Noll-Hussong

**Affiliations:** ^1^Department of Psychiatry and Psychotherapy, Saarland University Medical Center, Homburg, Germany; ^2^Psychosomatic Medicine and Psychotherapy, Saarland University Medical Center, Homburg, Germany; ^3^Department of Psychiatry and Psychotherapy, Medical Center – University of Freiburg, Faculty of Medicine, University of Freiburg, Freiburg, Germany; ^4^Center for Basics in NeuroModulation, Faculty of Medicine, University of Freiburg, Freiburg, Germany

**Keywords:** specific phobia, functional neuroimaging, augmented reality, virtual reality, psychotherapy, spider phobia, arachnophobia, neuroscience

## Abstract

Recent fMRI studies on specific animal phobias, particularly spider phobia (arachnophobia), have identified a large variety of specific brain regions involved in normal and disturbed fear processing. Both functional and structural brain abnormalities have been identified among phobic patients. Current research suggests that both conscious and subconscious fear processing play a crucial role in phobic disorders. Cognitive behavioral therapy has been identified as an effective treatment for specific phobias and has been associated with neuroplastic effects which can be evaluated using current neuroimaging techniques. Recent research suggests that new approaches using virtual (VR) or augmented reality (AR) tend to be similarly effective as traditional “*in vivo*” therapy methods and could expand treatment options for different medical or individual scenarios. This narrative review elaborates on neural structures and particularities of arachnophobia. Current treatment options are discussed and future research questions are highlighted.

## Introduction

Animal phobia is a type of specific phobia, which is classified under the Anxiety Disorders section of the DSM-5 (1). To meet the DSM-5 criteria for specific phobia, individuals have to show a marked and disproportionate fear or anxiety reaction when exposed to the phobic stimuli (i.e., animals like spiders). This fear or anxiety reaction may result in a full or limited panic attack. Due to fear or anxiety, individuals with specific phobia take significant steps to avoid the feared stimulus. Additionally, the fear and anxiety of a specific stimulus causes clinically significant distress or impairment in social, occupational, and in other relevant areas of function. Symptoms should last for at least 6 months. Individuals with specific phobias, who present without other comorbid diagnoses, are often seen in non-medical mental health settings. However, in medical-clinical settings, specific phobias are rarely seen without comorbid psychopathology, as they are frequently associated with other mental disorders, especially anxiety and depressive disorders (1). In fact, the median lifetime prevalence of specific phobia was estimated to be 7.2% with substantial variation between countries and sites (2). Most studies provide evidence for a higher risk to develop a specific phobia in females. Among the specific phobias, the prevalence of animal phobia is ranging among the most prevalent subtypes (with fear of heights). In this focused narrative review, we first elaborate on the neural structures involved in phobia before mentioning mental and neural processes, as well as structural and physiological changes. We continue by reviewing subconscious fear processing and cognitive behavioral therapy for arachnophobia, followed by a section of VR-AR based treatment options.

## Neural Structures Involved in Phobia

**Figure 1:**
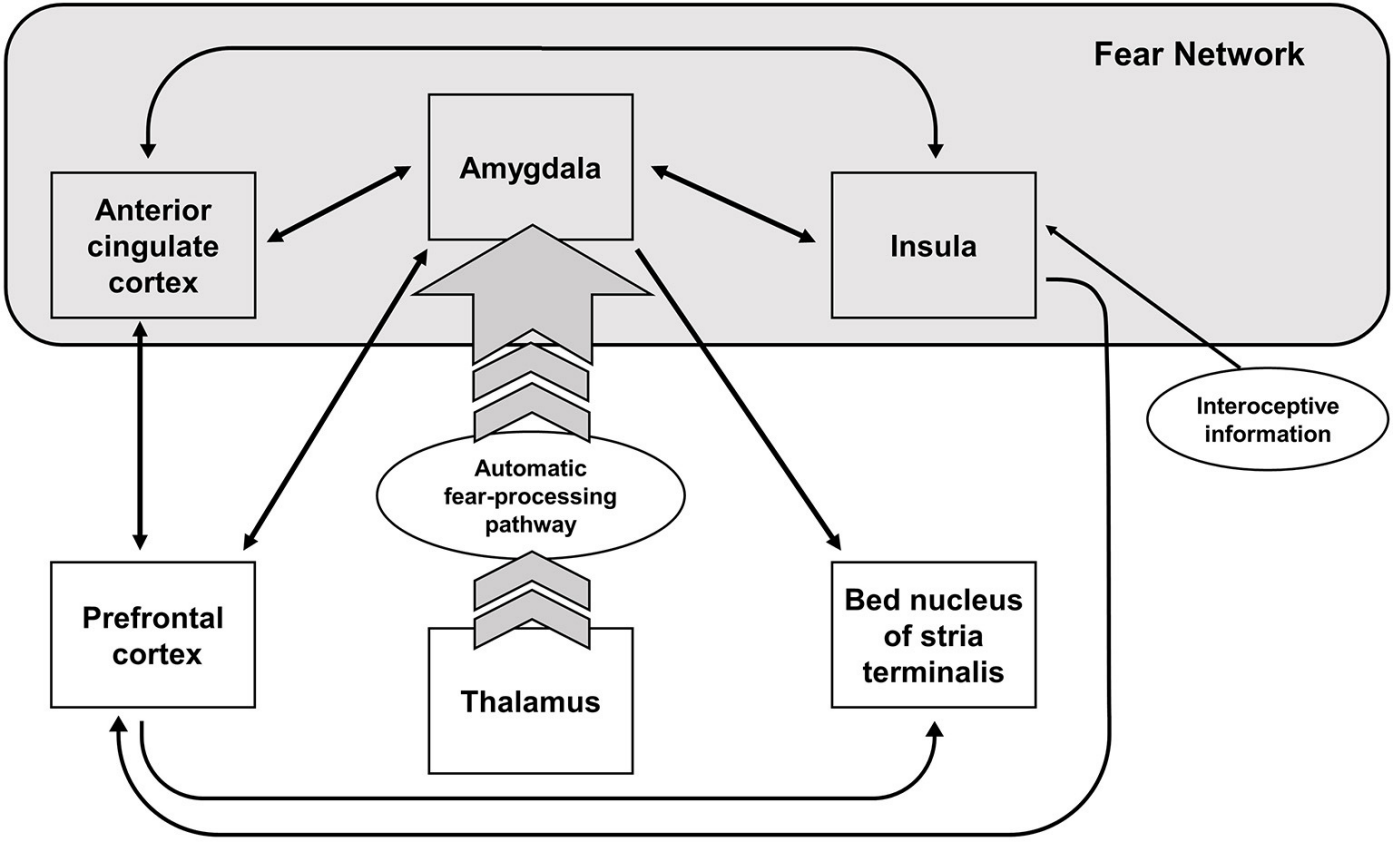
Neural structures involved in fear processing. Arrows indicate the direction of the activation.

Early neuroimaging studies of fear conditioning, as well as more recent experiments, have found evidence of an activation of the amygdala, the anterior cingulate cortex, and the insula—when healthy participants are confronted with conditioned fear-evoking stimuli independent of the task design (3). These structures are commonly referred to as the “fear network,” which is activated during the acquisition phase of fear-conditioning or corresponding paradigms, and shows stronger activation during extinction learning (4). The amygdala, an important structure for reward learning (5), processing of socially and emotionally relevant stimuli (6, 7), and fear acquisition (8), shows stronger activation to conditioned vs. unconditioned fear-evoking stimuli in healthy controls [e.g., (9)]. However, a more general approach assumes a state value-determining function of the amygdala (5, 10). According to this approach, the amygdala calculates and holds a continuously updated representation of value (5). Additionally, studies suggest that the amygdala is also involved in extinction learning in conjunction with the prefrontal cortex (PFC) (11). Apart from the amygdala, the anterior cingulate cortex and the insula are activated during fear conditioning. The insula, responsible for sensorimotor processing, socio-emotional processing, as well as higher cognitive functions, also holds representations of aversive body states (especially the left insular cortex) (12, 13). The anterior insula receives input from the amygdala and projects to the amygdala in turn. The main function of the anterior insula is thought to be the integration of internal states, whereas the posterior insula is responsible somato-visceral integration (14). Thus, the insula might integrate the somato-visceral sensations from anxiety inducing stimuli with the aversive stimuli in the environment (15). A comprehensive overview of the brain regions involved in the fear network can be seen in Figure 1.

Resting state fMRI paradigms suggest that the anterior cingulate cortex (ACC), as well as limbic- and hypothalamus- related areas, is functionally connected to areas responsible for affective processing (16). Although not all anxiety-related disorders (e.g., PTSD) show an increased activation of the subgenual ACC or the anterior middle cingulate cortex (17), e.g., patients suffering from social-anxiety disorder or specific phobias demonstrate an increased activity of the pregenual ACC and middle cingulate cortex in comparison to healthy controls (12, 17). In their meta-analysis, Etkin and Wagner (17) report hypoactivation in the insula and amygdala for PTSD patients, but no deactivations could be observed in specific phobia. Similarly, no hypoactivation in specific phobia was observed in the rostral anterior cingulate cortex, the ventromedial prefrontal cortex, the dorsal anterior cingulate cortex, or the thalamus. However, a more recent review reports evidence for hypoactivity in phobic patients in ventral, dorsomedial and dorsolateral prefrontal areas compared to healthy controls (15). These prefrontal areas are functionally connected to the amygdala. The dorsal system comprising the dorsolateral prefrontal cortex (DLPFC) and dorsomedial prefrontal cortex (DMPFC) might be related to the regulation of affective states, whereby a stronger activation is coupled with limbic inhibition (18–20). Altogether, these findings suggest a deficient regulatory mechanism of affective states in individuals suffering from specific phobia in comparison to healthy controls.

In general, current fMRI research studies indicate that a large array of brain regions are involved in both normal and phobic fear processing; however, the activation in phobic states has been found to be stronger (21). The stronger activation in phobic patients includes increased neural, electrodermal, pupillary, and subjective reactions, as well as stronger activated neural structures, compared to non-phobic individuals. For instance, when confronted with the feared stimulus, individuals with spider phobia show increased activation of different brain regions, particularly in areas related to visuo-attentional processing (occipital and parietal regions; ventral visual pathway), emotional processing (amygdala, pulvinar nucleus of the thalamus), representations of aversive body states (ACC, insula, specifically left insular cortex), and flight behavior processing (premotor areas) (22–24).

Interestingly, individuals suffering from spider phobia compared to non-phobic individuals show an increased fear response pattern in the mid-insula, dorsal anterior cingulate, and the ventrolateral PFC that grows linearly with perceived danger. Thus, these brain regions seem to hold a quantitative representation of the exaggerated fear response (25). While most of the regions mentioned above are involved in normal fear processing, the activation of the supplementary motor area (SMA) seems to be unique in specific phobias (26).

As mentioned above, a predominantly known key structure in fear processing is the amygdala, which usually shows hyperactivity in individuals with phobia (23). Increased activity of the amygdala is due to an exaggerated input from a fast subcortical pathway and can be reduced by glucocorticoid administration (27). An extrageniculostriate pathway has been suggested as the main pathway responsible for fear processing, which connects the thalamus and the amygdala (23). In contrast to common fear theories, which postulate that the activation of the amygdala is independent from attention when perceiving threatening stimuli, recent research shows that amygdala activation in response to very briefly presented cues is not independent from attention processes in patients with phobia (28). However, the amygdala response to phobic stimuli among individuals with phobia is a stronger but briefer one as compared to healthy controls, suggesting that it might be responsible for the rapid onset of phobic disorders (29). Additionally, the increased activity in the amygdala in patients with phobia does not only occur when they are confronted with phobic stimuli, but also as a response to generally repulsive and threatening stimuli (26). This suggests that increased activity in the amygdala may occur as a result of detection of motivationally relevant stimuli in general, whereas the activation of the insula and supplementary motor area seem to be superficially connected to the processing of phobic stimuli (26, 30). The attenuating effect of glucocorticoid administration on the fear response of the amygdala among individuals with phobia might be due to the fact that high glucocorticoid levels weaken traumatic and fearful memory traces, which are assumed to play a crucial role in phobic disorders and therefore facilitate the extinction of phobic fear (31). It has been recently shown that cortisol administration was able to alter the activity of the salience network to a level that was comparable to that found in healthy controls (32). In addition, high endogenous cortisol levels, which physiologically peak in the morning, strengthen the effect of exposure therapy in spider phobia compared to lower cortisol levels in the afternoon (33).

## Mental and Neural Processes Involved in Fear Processing

While structures of the network mentioned above are involved in fear processing in general, their specific roles seem to be more complex. The temporo-spatial activation of different brain areas in spider phobia can especially be linked to different emotion regulation deficiencies. While automatic emotion regulation deficiencies seem to be reflected by increased activity in the insula and reduced activity in the ventromedial PFC, effortful emotion regulation deficits are reflected in an enhanced activity in medial PFC areas (34). Different neural processes have also been found for phasic fear in comparison to sustained anxiety (35). Phasic fear is defined as an apprehensive state that accumulates rapidly and diminishes if the threatening stimulus is removed (36). While phasic fear was found to be associated with amygdala activity, sustained anxiety was identified to be related to activity in the bed nucleus of stria terminalis (BNST), the ACC, and the insula among spider phobic patients compared to healthy controls (35). When comparing brain activation patterns in different types of phobias, several studies show very similar immediate responses but differences in the sustained response, which reflects the cross-linked mental processes behind anxiety disorders (12, 22). These findings correspond to fMRI studies on anticipatory fear of phobic stimuli. Straube et al. (37) showed that patients with phobia showed increased activity in the ACC, insula, thalamus, visual areas (i.e., fusiform gyrus), and BNST in comparison to healthy controls. This suggests that ACC regions and the anterior medial PFC seem to be related to the severity of the phobic disorder (37). These findings support the idea of the amygdala being related to automatic fear processing, while the insula, ACC, and dorsomedial PFC seem to be related to direct fear processing which requires attentional processes (37). In sum, these data highlight the dynamic relationship underlying neural processes involved in fear reactions, which is consistent with the finding that both visual and linguistic stimuli (phobia related words) are able to activate the aforementioned fear-related brain regions, supporting the idea of integrating neural networks for the processing of threatening stimuli (38).

## Structural and Physiological Changes

Both functional and structural abnormalities like thinning of the right ACC can be found in patients with spider phobia (39). The size of the amygdala is negatively correlated to severity of symptoms among phobic patients (40). However, it remains unclear whether the structural changes are a causal or a consequence of the symptoms.

Phobias can not only be associated to neural abnormalities but also associated with physiological changes. When exposed to a phobic stimulus, patients with phobia show somatic response patterns or “markers” which differ from healthy controls, such as greater heart rate amplitudes and hypervigilance, as well as increased pupillary dilatation, electrodermal response, and self-reported affect (21, 41). Although some of these markers do not reflect a general tendency for anxiety, they are stimuli-dependent when comparing different types of phobias in fMRI studies (41).

The evidence for sex differences in activation patterns as a response to phobic stimuli is still not entirely clear. There is some evidence that phobic activation patterns may differ between males and females. These differences might be due to different attention styles and self-control strategies (42) as well as more pronounced and repetitive negative thoughts in females compared to males (43). Further support is based on epidemiological studies showing a higher frequency of phobic disorders in young female patients (44).

## Biopsychosocial Theories of Phobias

Past research has relied on Seligman's theory of prepared learning (45, 46) to explain the nature of fear and the rise of phobias. However, more recent research shows again that the underlying mechanisms might be more differentiated. For instance, animal phobias (i.e., spider phobia) have been found to be more robust and less susceptible to social influence compared to social fears (47). Hence, in order to explain phobic disorders, a bio-psycho-social theory combining particularly genetic and cultural factors seems be more useful. For example, the heritability in animal fear is estimated to be 45% (48).

Studies on the biological origin of fear and phobic reactions have highlighted the role of structural differences in anxiety sensitivity, such as the inclination to interpret physical symptoms as potentially threatening. Anxiety sensitivity is found to be correlated to right anterior insular cortex size (volume and thickness) in patients with spider phobia (49). Other structural regions that are involved in emotional and attentional processing are also involved in the detection of body states and motor behavior (22–24).

Not surprisingly, a crucial part in the neurobiological understanding of phobic disorders is the involvement of the amygdala. While common fear theories postulate that its activation is independent from attentional processes (23), others postulate that the amygdala's activation is influenced by distractors (28), suggesting that attention is needed for its activation. The strong and brief activation of the amygdala when confronted with distressing stimuli is hypothesized to be responsible for the rapid onset of phobic disorders (29), suggesting that minimal exposure with phobic objects is sufficient for the onset of the disorder. Other research proposes a more general overactivation of the area, which does not seem to be connected to specific stimuli, but rather to generally motivational relevant stimuli (26). Altogether, the amygdala has been identified as a key region of interest when trying to understand phobic disorders; however, the exact mechanisms remain to be understood.

## Subconscious Fear Processing

Phobic stimuli are assumed to have greater affective salience in patients with phobia and therefore gain a more rapid access to awareness. Schmack et al. (50) demonstrated that the activation in orbitofrontal and ventral visual areas (higher-level cortical areas) as a response to the presence of phobic stimuli could be crucial for the affective salience of subjective invisible object stimuli (suppressed from view). Further research has shown that presentation of subliminal stimulus activates fear regions in spider phobia (right amygdala) while deactivating conscious fear processing, suggesting that the threat is computed even before the stimulus is processed consciously (51–53). While some studies suggest that amygdala activation in spider phobia is robust to distraction tasks requiring the participant's attention (54), other studies have found that amygdala activation appears to decrease in healthy controls when performing distraction tasks (54–56). Overall, there is some evidence that phobic reactions can occur outside an individual's awareness, however, the role of distraction in this process is not clear.

## Phobic Cognition Biases

The *covariation bias* is a common bias that occurs among individuals with phobias. There is evidence suggesting that individuals with phobias tend to overestimate the association between the feared stimulus and aversive consequences (57). Recent research suggests that the covariation bias may be enhanced by simultaneous activity in senso-motoric and visual cortices, while the right PFC has been found to reduce the bias (57). In addition to the covariation bias, patients with phobia tend to show an encounter expectancy bias which means that they overestimate the likelihood that they will encounter a feared object (58). Recent fMRI studies suggest that the encounter expectancy bias appears to be related to deficits in cognitive control and contextual integration (lateral PFC, precuneus, and visual cortex) (58). Furthermore, a dysfunctional circuit of greater rostral ACC activation in response to phobic related stimuli compared to neutral stimuli was found in subjects with animal phobia, but not in healthy controls, suggesting attention abnormalities (59). Other studies indicate that amygdala activation depends on attention allocation when the stimuli are briefly presented (28), which might contribute to the behaviorally observable attention bias in anxious individuals (60).

## Treatment of Spider Phobia: Effects of Cognitive Behavioral Therapy

Although a variety of treatment options for specific phobia exist, this review focuses on CBT and new approaches based on CBT-techniques, as they are regarded to be clinically the most promising evidence-based therapeutic procedures so far (61). However, a variety of other treatment options exist, including (but are not limited to) psychopharmacotherapy (e.g., SSRI or benzodiazepine medication), hypnotherapy, cognitive therapy, psychodynamic psychotherapy, imaginal or virtual reality exposure, augmented or *in vivo*-exposure.

Ongoing research on various modalities of psychological treatments for mental health disorders strongly suggests that problem-oriented psychotherapy can reliably modulate the neurophysiological and neurochemical processes in brain regions responsible for perception, movement, pain, and emotion processing (62, 63). Functional MRI-resting state studies among individuals with spider phobia show that cerebral blood flow in fear-related brain regions is reduced after cognitive behavioral interventions (64). This effect was not only found for the anticipatory fear, but also for fear processing after the presentation of spider pictures in the scanner (64). Some fMRI studies have shown a significant pre to post decrease in amygdala, insula, and ACC hyperactivity among individuals with phobia after an intensive one-session exposure therapy was provided (23). In addition, the dorsolateral PFC and the parahippocampal gyrus have been found to be hyperactive among individuals with spider phobia prior to receiving therapy, which might reflect deficiencies in metacognitive strategies when confronted with a spider on the one hand and fear memory on the other; however, these effects disappeared after successful therapy (63). Eye-tracking studies show that patients use maladaptive coping skills (i.e., visual avoidance) when faced with phobic stimuli whilst their fear circuit is activated and experiencing a threat (65). In comparison, healthy controls show more effective coping skills when paying visual attention to potentially dangerous stimuli, such as looking at them for a longer period of time; this might help to downregulate cognitive evaluations of risk (65). This might also be the case among successfully treated individuals who learned to effectively cope with perceived threats by reducing avoidance. Furthermore, fMRI studies have revealed that the long-term positive effects of CBT may be due to increased activation of the orbitofrontal cortex (OFC), which is involved both in emotional-related learning and neural inhibition (66).

CBT has been found to effectively treat specific phobias in most, but not all cases. Recent research identified different neural mechanisms for automatic and elaborated responses to threat (51). For instance, hyperactivation in the right amygdala and fusiform gyrus were found to rise when subliminal phobic stimuli were shown, an effect which did not disappear after CBT, suggesting that CBT might only influence conscious fear responses. Moreover, Zilverstand et al. (67) found that neurofeedback facilitates anxiety regulation in spider phobia when comparing a neurofeedback-group to a group with no specific strategies *via* fMRI. The neurofeedback-participants showed significantly lower anxiety levels at the end of the neurofeedback training as well as a down-regulation of the insular region. Neurofeedback was provided in response to activation in the left dorsolateral PFC and right insula, which was used as a marker for successful regulation of the phobic fear (67). Similar effects were observed for the hyperactivity of the insula and ACC when confronted with phobic stimuli, as this hyperactivity disappeared after successful CBT (95).

Another crucial contributing factor to the outcomes of CBT treatment in phobia might be that fear-relevant emotions can modulate learning processes and change activity in frontostriatal and medial temporal lobes, which are associated with specific learning processes (68). In addition, research shows that the effects of CBT can be enhanced by the administration of D-cycloserine, which is known to enforce the activations in the fear relevant brain regions (69); this suggests that high levels of acute activation in the fear regions predict better therapy outcomes. For a comprehensive overview of the present state of literature on neural networks predicting psychotherapy response in anxiety disorders, see Lueken et al. (70). Considering the mentioned gender differences in fear processing, recent research also suggested that that progesterone could be a useful pharmacological adjunct to cognitive therapy (71).

## New Approaches: Virtual and Augmented Reality in Phobia Treatment

Although *in-vivo* exposure provides an excellent treatment option, patients are often reluctant to leverage the treatment options. Therapists often avoid the logistic difficulties that come with *in-vivo*-exposure therapies (e.g., time-consuming therapy session, catching, and keeping the spiders). In addition, spider-phobic individuals can be reluctant to confront their fears in reality. Hence, treatment options using virtual or augmented realities might be an interesting alternative from different viewpoints. Augmented Reality (AR) is a new approach to treatment that allows enriching real-life environments with virtual elements and has been successfully applied in various settings including treatment of mental health disorders (72). Advantages of AR in the treatment of psychological disorders include the adaptability to individual needs, as well as its accessibility and ecological validity, such that it can be used in personally relevant, real life settings (72). Furthermore, rare scenarios (e.g., special spider moving behaviors) can be administered repeatedly and consequentially the threatening content of the simulated spider can be varied more easily. Early research on AR and its use to treat specific phobia (especially small animal phobia) provides an insight on the feasibility of this new approach (73–77). Newer studies demonstrate equivalent treatment efficacy between augmented reality based and *in-vivo* exposure therapies (78–80). Moreover, the safety and usability of augmented reality systems in clinical samples has been demonstrated [e.g., (81)].

Virtual Reality (VR), in contrast, describes completely simulated, artificial environments—an approach which has recently gained popularity to treat psychiatric diseases, in particular anxiety disorders (82). The success of VR therapy approaches is supported by various research reports showing good effects for adults and children (82, 83). Patients with specific phobias prefer a virtual treatment-option over *in-vivo*-exposure and have fewer reservations (84). Several trials have demonstrated efficacy of VR-based exposure treatment in spider phobia when compared to a control condition (85, 86). Furthermore, non-inferiority of virtual reality exposure therapy for small animal or spider phobia was demonstrated in comparison to an *in-vivo* exposure therapy, particularly after 3 (87, 88) and 12 months (88). Research using fMRI to examine brain activation while participants explore VR environments shows that identical anxiety-related brain regions are activated as with real life photographs (89). Some studies suggest a potentially beneficial augmentative effect of non-invasive brain stimulation such as intermittent Theta Burst Stimulation (iTBS) over the left prefrontal cortex on a VR challenge in spider phobia, supporting the relevance of the prefrontal network in phobia treatment [e.g., (86, 90)].

## Discussion

While technically new approaches promise many advantages in the field of treating natural object phobias (such as spider phobia), one can question if unpredictable nature elements like a real threat can be completely replaced by artificial elements at this stage. More research has to be conducted to assess the similarity between simulated human behavior and real-life behavior. Concerns regarding addictive behavior in virtual reality have also been raised (83). Because a sense of immersion is important in virtual reality therapy, new approaches promise—besides further technical improvements—the integration of tactile simulation in conjunction to the simulated visual experience.

Altogether, meta-analyses estimating the outcome and effectiveness of VR therapy state that VR-based psychological procedures appear to be equivalent to *in-vivo* exposure therapy, and superior to no treatment, and comes along with several advantages such as accessibility [e.g. (91, 92)]. However, there is currently no evidence that VR therapy could be superior to conventional face-to-face therapy methods with a real therapist. Additionally, more light has to be shed on both possible side effects like cybersickness (symptoms like vertigo, nausea, and headache) and methods to circumvent these have to be established (83, 93, 94). Future studies should also focus on improving the immersive experience for the user, e.g., by combining the simulated visual experience with tactile or auditory information. To the best of our knowledge, no study to date includes the auditory input in the simulated environment, e.g., when a particularly large spider moves over a surface. In addition, the therapy systems available to date require input from the therapist. In order to increase accessibility of exposure therapy interventions, a standalone therapy system is favorable. Last not least, a future challenge will be the smooth real time interaction between the simulated phobic object and the hands of the participants.

The aim of this focused review was to provide an overview and summarize recent findings in the field of specific phobic disorders, especially spider phobia. A particular emphasis was drawn on brain circuits and neural networks associated with spider phobia and treatment using cognitive behavioral therapy (“gold standard”) and emerging approaches including virtual or augmented reality.

Functional MRI has proven to be a strong research tool to investigate the neural basis for both normal and phobic fear. Accordingly, research over the past decade has been able to identify a large array of brain regions associated with fear processing: not only those involved in emotional and attentional processing, but also for the detection of body states and motor behavior. While the amygdala seems to be mainly involved in automatic fear processing, albeit not fully independent of attentional processes, other structures as the insula, ACC, and dorsomedial PFC appear to be directly related to fear processing requiring attentional resources (37). As a consequence, further research is mandatory for a better understanding of the underlying mechanisms and the neural bases of spider phobia for both predicting and potentially enhancing therapy outcome.

Cognitive behavioral therapy has been proven as a very effective clinical treatment and its effects can be observed even at a neural level (62). The newly emerging approaches such as virtual and augmented reality appear to show similar effects in comparison to *in-vivo* exposure therapy while increasing the accessibility and acceptance of treatment opportunities and supporting therapy-resistant patients (72, 83). Virtual and augmented reality-based therapy can thus already provide an excellent add-on option to the usual treatment. However, further research in VR/AR-based therapies is mandatory, in particular with a focus on improvement in feasibility and prevention and management of the immanent side effects of VR-based therapies.

## Author Contributions

JH and AR substantially contributed to the conception of the work and drafting the first version of the document. UM, NM, KD, MR, and MN-H contributed to the analysis and critically revising the document as well as providing final approval of the version to be published.

## Conflict of Interest

KD is a member of Janssen Pharmaceuticals Steering Committee Neurosciences. The remaining authors declare that the research was conducted in the absence of any commercial or financial relationships that could be construed as a potential conflict of interest.

## Publisher's Note

All claims expressed in this article are solely those of the authors and do not necessarily represent those of their affiliated organizations, or those of the publisher, the editors and the reviewers. Any product that may be evaluated in this article, or claim that may be made by its manufacturer, is not guaranteed or endorsed by the publisher.

## References

[1] American Psychiatric Association. Diagnostic and Statistical Manual of Mental Disorders: DSM-5. 5th ed. Washington, DC: Author (2013).

[2] Eaton WW Bienvenu J Miloyan B. Specific phobias. Lancet Psychiatry. (2018) 5:678–86. 10.1016/S2215-0366(18)30169-X 30060873 PMC7233312

[3] Sehlmeyer C Schöning S Zwitserlood P Pfleiderer B Kircher T Arolt V. Human fear conditioning and extinction in neuroimaging: a systematic review. PLoS ONE. (2009) 4:e5865. 10.1371/journal.pone.0005865 19517024 PMC2692002

[4] Holzschneider K Mulert C. Neuroimaging in anxiety disorders. Dialogues Clin Neurosci. (2011) 13:453–61. 10.31887/DCNS.2011.13.4/kholzschneider 22275850 PMC3263392

[5] Morrison SE Salzman CD. Re-valuing the amygdala. Curr Opin Neurobiol. (2010) 20:221–30. 10.1016/j.conb.2010.02.007 20299204 PMC2862774

[6] Adolphs R. What does the amygdala contribute to social cognition? Ann. N Y Acad Sci. (2010) 1191:42–61. 10.1111/j.1749-6632.2010.05445.x 20392275 PMC2871162

[7] Sabatinelli D Fortune EE Li Q Siddiqui A Krafft C Oliver WT. Emotional perception: meta-analyses of face and natural scene processing. Neuroimage. (2011) 54:2524–33. 10.1016/j.neuroimage.2010.10.011 20951215

[8] Büchel C Dolan RJ. Classical fear conditioning in functional neuroimaging. Curr Opin Neurobiol. (2000) 10:219–23. 10.1016/S0959-4388(00)00078-7 10753800

[9] Büchel C Morris J Dolan RJ Friston KJ. Brain systems mediating aversive conditioning: an event-related FMRI study. Neuron. (1998) 20:947–57. 10.1016/S0896-6273(00)80476-6 9620699

[10] Zheng J Anderson KL Leal SL Shestyuk A Gulsen G Mnatsakanyan L Vadera S. Amygdala-hippocampal dynamics during salient information processing. Nat Commun. (2017) 8:14413. 10.1038/ncomms14413 28176756 PMC5309795

[11] Delgado MR Nearing KI Ledoux JE Phelps EA. Neural circuitry underlying the regulation of conditioned fear and its relation to extinction. Neuron. (2008) 59:829–38. 10.1016/j.neuron.2008.06.029 18786365 PMC3061554

[12] Caseras X Murphy K Mataix-Cols D López-Solà M Soriano-Mas C Ortriz H. Anatomical and functional overlap within the insula and anterior cingulate cortex during interoception and phobic symptom provocation. Hum Brain Mapp. (2013) 34:1220–9. 10.1002/hbm.21503 22162203 PMC6869871

[13] Uddin LQ Nomi JS Hebert-Seropian B Ghaziri J Boucher O. Structure and function of the human insula. J Clin Neurophysiol. (2017) 34:300–6. 10.1097/WNP.0000000000000377 28644199 PMC6032992

[14] Paulus MP Stein MB. An insular view of anxiety. Biol Psychiatry. (2006) 60:383–7. 10.1016/j.biopsych.2006.03.042 16780813

[15] Del Casale A Ferracuti S Rapinesi C Serata D Piccirilli M Savoja V. Functional neuroimaging in specific phobia. Psychiatry Res Neuroimaging. (2012) 202:181–97. 10.1016/j.pscychresns.2011.10.009 22804970

[16] Stevens FL Hurley RA Hurley RA Hayman LA Taber KH. Anterior cingulate cortex: unique role in cognition and emotion. J Neuropsychiatry Clin Neurosci. (2011) 23:121–5. 10.1176/jnp.23.2.jnp121 21677237

[17] Etkin A Wager TD. Functional neuroimaging of anxiety: a meta-analysis of emotional processing in PTSD, social anxiety disorder, and specific phobia. Am J Psychiatry. (2007) 164:1476–88. 10.1176/appi.ajp.2007.07030504 17898336 PMC3318959

[18] Ochsner K Gross J. The cognitive control of emotion. Trends Cogn Sci. (2005) 9:242–9. 10.1016/j.tics.2005.03.010 15866151

[19] Phillips ML Young AW Senior C Brammer M Andrew C Calder AJ. A specific neural substrate for perceiving facial expressions of disgust. Nature. (1997) 389:495–8. 10.1038/39051 9333238

[20] Phelps EA. Emotion and cognition: insights from studies of the human amygdala. Annu Rev Psychol. (2006) 57:27–53. 10.1146/annurev.psych.56.091103.070234 16318588

[21] Schaefer HS Larson CL Davidson RJ Coan JA. Brain, body, and cognition: neural, physiological and self-report correlates of phobic and normative fear. Biol Psychol. (2014) 98:59–69. 10.1016/j.biopsycho.2013.12.011 24561099 PMC4251669

[22] Caseras XD Mataix-Cols MV Trasovares M López-Solà H Ortriz J Pujol C. Dynamics of brain responses to phobic-related stimulation in specific phobia subtypes. Euro J Neurosci. (2010) 32:1414–22. 10.1111/j.1460-9568.2010.07424.x 20950283

[23] Goossens L Sunaert S Peeters R Griez EJL Schruers KRJ. Amygdala hyperfunction in phobic fear normalizes after exposure. Biol Psychiatry Stress Anxiety Dev Ther Perspect. (2007) 62:1119–25. 10.1016/j.biopsych.2007.04.024 17706612

[24] Scharmüller W Leutgeb V Schäfer A Köchel A Schienle A. Source localization of late electrocortical positivity during symptom provocation in spider phobia: an SLORETA study. Brain Res. (2011) 1397:10–8. 10.1016/j.brainres.2011.04.018 21600565 PMC3119789

[25] Zilverstand A Sorger B Kaemingk A Goebel R. Quantitative representations of an exaggerated anxiety response in the brain of female spider phobics—a parametric FMRI study. Hum Brain Mapp. (2017) 38:3025–38. 10.1002/hbm.23571 28321945 PMC6867041

[26] Schienle A Schafer A Walter B Stark R Vaitl D. Brain activation of spider phobics towards disorder-relevant, generally disgust- and fear-inducing pictures. Neurosci Lett. (2005) 388:1–6. 10.1016/j.neulet.2005.06.025 16046064

[27] Nakataki M Soravia LM Schwab S Horn H Dierks T Strik W. Glucocorticoid administration improves aberrant fear-processing networks in spider phobia. Neuropsychopharmacology. (2017) 42:485–94. 10.1038/npp.2016.207 27644128 PMC5399241

[28] Alpers GW Gerdes ABM Lagarie B Tabbert K Vaitl D Stark R. Attention and amygdala activity: an FMRI study with spider pictures in spider phobia. J Neural Transm. (2009) 116:747–57. 10.1007/s00702-008-0106-8 18726545

[29] Larson CL Schaefer HS Siegle GJ Jackson CAB Anderle MJ Davidson RJ. Fear is fast in phobic individuals: amygdala activation in response to fear-relevant stimuli. Biol Psychiatry. (2006) 60:410–7. 10.1016/j.biopsych.2006.03.079 16919528

[30] Wendt J Lotze M Weike AI Hosten N Hamm AO. Brain activation and defensive response mobilization during sustained exposure to phobia-related and other affective pictures in spider phobia. Psychophysiology. (2008) 45:205–15. 10.1111/j.1469-8986.2007.00620.x 17995911

[31] de Quervain DJ-F Margraf J. Glucocorticoids for the treatment of post-traumatic stress disorder and phobias: a novel therapeutic approach. Eur J Pharmacol. (2008) 583:365–71. 10.1016/j.ejphar.2007.11.068 18275950

[32] Soravia LM Schwab S Weber N Nakataki M Wiest R Strik W. Glucocorticoid administration restores salience network activity in patients with spider phobia. Depress Anxiety. (2018) 35:925–34. 10.1002/da.22806 30099829

[33] Lass-Hennemann J Michael T. Endogenous cortisol levels influence exposure therapy in spider phobia. Behav Res Ther. (2014) 60:39–45. 10.1016/j.brat.2014.06.009 25051297

[34] Hermann A Schäfer A Walter B Stark R Vaitl D Schienle A. Emotion regulation in spider phobia: role of the medial prefrontal cortex. Soc Cogn Affect Neurosci. (2009) 4:257–67. 10.1093/scan/nsp013 19398537 PMC2728632

[35] Münsterkötter AL Notzon S Redlich R Grotegerd D Dohm K Arolt V. Spider or no spider? Neural correlates of sustained and phasic fear in spider phobia. Depress Anxiety. (2015) 32:656–63. 10.1002/da.22382 26115440

[36] Davis M Walker DL Miles L Grillon C. Phasic vs sustained fear in rats and humans: role of the extended amygdala in fear vs anxiety. Neuropsychopharmacology. (2010) 35:105–35. 10.1038/npp.2009.109 19693004 PMC2795099

[37] Straube T Mentzel H-J Miltner WHR. Neural mechanisms of automatic and direct processing of phobogenic stimuli in specific phobia. Biol Psychiatry. (2006) 59:162–70. 10.1016/j.biopsych.2005.06.013 16139812

[38] Straube T Mentzel H-J Glauer M Miltner WHR. Brain activation to phobia-related words in phobic subjects. Neurosci Lett. (2004) 372:204–8. 10.1016/j.neulet.2004.09.050 15542241

[39] Linares IMP Jackowski AP Trzesniak CMF Arrais KC Chagas MHN Sato JR. Cortical thinning of the right anterior cingulate cortex in spider phobia: a magnetic resonance imaging and spectroscopy study. Brain Res. (2014) 1576:35–42. 10.1016/j.brainres.2014.05.040 24892191

[40] Fisler MS Federspiel A Horn H Dierks T Schmitt W Wiest R. Spider phobia is associated with decreased left amygdala volume: a cross-sectional study. BMC Psychiatry. (2013) 13:70. 10.1186/1471-244X-13-70 23442196 PMC3599010

[41] Michałowski JM Matuszewski J Drozdziel D Koziejowski W Rynkiewicz A Jednoróg K. (2017). Neural response patterns in spider, blood-injection-injury and social fearful individuals: new insights from a simultaneous EEG/ECG–FMRI study. Brain Imaging Behav. 11:829–45. 10.1007/s11682-016-9557-y 27194564

[42] Schienle A Scharmüller W Leutgeb V Schäfer A Stark R. Sex differences in the functional and structural neuroanatomy of dental phobia. Brain Struct Funct. (2013) 218:779–87. 10.1007/s00429-012-0428-z 22644919

[43] Graham BM Weiner S Li SH. Gender differences in avoidance and repetitive negative thinking following symptom provocation in men and women with spider phobia. Br J Clin Psychol. (2020) 59:565–77. 10.1111/bjc.12267 32955767

[44] Becker ES Rinck M Türke V Kause P Goodwin R Neumer S. Epidemiology of specific phobia subtypes: findings from the dresden mental health study. Euro Psychiatry. (2007) 22:69–74. 10.1016/j.eurpsy.2006.09.006 17157482

[45] Seligman M. On the generality of the laws of learning. Psychol Rev. (1970) 77:406–18. 10.1037/h0029790

[46] Seligman M. Phobias and preparedness. Behav Ther. (1971) 2:307–20. 10.1016/S0005-7894(71)80064-3

[47] Mallan KM Lipp OV Cochrane B. Slithering snakes, angry men and out-group members: what and whom are we evolved to fear? Cogn Emot. (2013) 27:1168–80. 10.1080/02699931.2013.778195 23556423

[48] Van Houtem CMHH Laine ML Boomsma DI Ligthart L van Wijk AJ De Jongh A. A review and meta-analysis of the heritability of specific phobia subtypes and corresponding fears. J Anxiety Disord. (2013) 27:379–88. 10.1016/j.janxdis.2013.04.007 23774007

[49] Rosso IM Makris N Britton JC Price LM Gold AL Zai D. Anxiety sensitivity correlates with two indices of right anterior insula structure in specific animal phobia. Depress Anxiety. (2010) 27:1104–10. 10.1002/da.20765 21132846 PMC3010373

[50] Schmack K Burk J Haynes J-D Sterzer P. Predicting subjective affective salience from cortical responses to invisible object stimuli. Cereb Cortex. (2016) 26:3453–60. 10.1093/cercor/bhv174 26232987

[51] Lipka J Hoffmann M Miltner WHR Straube T. Effects of cognitive-behavioral therapy on brain responses to subliminal and supraliminal threat and their functional significance in specific phobia. Biol Psychiatry Anxiety Disord. (2014) 76:869–77. 10.1016/j.biopsych.2013.11.008 24393393

[52] Siegel P Warren R Wang Z Yang J Cohen D Anderson JF. Less is more: neural activity during very brief and clearly visible exposure to phobic stimuli. Hum Brain Mapp. (2017) 38:2466–81. 10.1002/hbm.23533 28165171 PMC5385151

[53] Siegel P Wang Z Murray L Campos J Sims V Leighton E. Brain-based mediation of non-conscious reduction of phobic avoidance in young women during functional MRI: a randomised controlled experiment. Lancet Psychiatry. (2020) 7:971–81. 10.1016/S2215-0366(20)30285-6 33069319

[54] Straube T Lipka J Sauer A Mothes-Lasch M Miltner WHR. Amygdala activation to threat under attentional load in individuals with anxiety disorder. Biol Mood Anxiety Disord. (2011) 1:12. 10.1186/2045-5380-1-12 22738024 PMC3384227

[55] Bishop SJ Jenkins R Lawrence AD. Neural processing of fearful faces: effects of anxiety are gated by perceptual capacity limitations. Cereb Cortex. (2007) 17:1595–603. 10.1093/cercor/bhl070 16956980

[56] Straube T Weiss T Mentzel H-J Miltner WHR. Time course of amygdala activation during aversive conditioning depends on attention. Neuroimage. (2007) 34:462–9. 10.1016/j.neuroimage.2006.08.021 17070072

[57] Wiemer J Pauli P. Enhanced functional connectivity between sensorimotor and visual cortex predicts covariation bias in spider phobia. Biol Psychol. (2016) 121:128–37. 10.1016/j.biopsycho.2016.01.007 26805508

[58] Aue T Hoeppli ME Piguet C Hofstetter C Rieger SW Vuilleumier P. Brain systems underlying encounter expectancy bias in spider phobia. Cogn Affect Behav Neurosci. (2015) 15:335–48. 10.3758/s13415-015-0339-6 25694215

[59] Britton JC Gold AL Deckersbach T Rauch SL. Functional MRI study of specific animal phobia using an event-related emotional counting stroop paradigm. Depress Anxiety. (2009) 26:796–805. 10.1002/da.20569 19434621 PMC2792204

[60] Bar-Haim Y Lamy D Pergamin L Bakermans-Kranenburg MJ van IJzendoorn MH. Threat-related attentional bias in anxious and nonanxious Individuals: a meta-analytic study. Psychol Bull. (2007) 133:1–24. 10.1037/0033-2909.133.1.1 17201568

[61] Choy Y Fyer AJ Lipsitz JD. Treatment of specific phobia in adults. Clin Psychol Rev. (2007) 27:266–86. 10.1016/j.cpr.2006.10.002 17112646

[62] Beauregard M. Effect of mind on brain activity: evidence from neuroimaging studies of psychotherapy and placebo effect. Nord J Psychiatry. (2009) 63:5–16. 10.1080/08039480802421182 19023697

[63] Paquette V Lévesque J Mensour B Leroux J-M Beaudoin G Bourgouin P. ‘Change the mind and you change the brain': effects of cognitive-behavioral therapy on the neural correlates of spider phobia. Neuroimage. (2003) 18:401–9. 10.1016/S1053-8119(02)00030-7 12595193

[64] Soravia LM Orosz A Schwab S Nakataki M Wiest R Federspiel A. CBT reduces CBF: cognitive-behavioral therapy reduces cerebral blood flow in fear-relevant brain regions in spider phobia. Brain Behav. (2016) 6:e00510. 10.1002/brb3.510 27688940 PMC5036433

[65] Aue T Hoeppli ME Piguet C Sterpenich V Vuilleumier P. Visual avoidance in phobia: particularities in neural activity, autonomic responding, and cognitive risk evaluations. Front Hum Neurosci. (2013) 7:194. 10.3389/fnhum.2013.00194 23754994 PMC3668156

[66] Schienle A Schäfer A Stark R Vaitl D. Long-term effects of cognitive behavior therapy on brain activation in spider phobia. Psychiatry Res Neuroimaging. (2009) 172:99–102. 10.1016/j.pscychresns.2008.11.005 19321317

[67] Zilverstand A Sorger B Sarkheil P Goebel R. FMRI neurofeedback facilitates anxiety regulation in females with spider phobia. Front Behav Neurosci. (2015) 9:148. 10.3389/fnbeh.2015.00148 26106309 PMC4458693

[68] Prince SE Thomas LA Kragel PA LaBar KS. Fear-relevant outcomes modulate the neural correlates of probabilistic classification learning. NeuroImage. (2012) 59:695–707. 10.1016/j.neuroimage.2011.07.027 21827859 PMC3195832

[69] Aupperle RL Hale LR Chambers RJ Cain SE Barth FX Sharp SC. An FMRI study examining effects of acute D-cycloserine during symptom provocation in spider phobia. CNS Spectr. (2009) 14:556–71. 10.1017/S1092852900024044 20095368

[70] Lueken U Zierhut KC Hahn T Straube B Kircher T Reif A. Neurobiological markers predicting treatment response in anxiety disorders: a systematic review and implications for clinical application. Neurosci Biobehav Rev. (2016) 66:143–62. 10.1016/j.neubiorev.2016.04.005 27168345

[71] Li SH Graham BM. Progesterone levels predict reductions in behavioral avoidance following cognitive restructuring in women with spider phobia. J Affect Disord. (2020) 270:1–8. 10.1016/j.jad.2020.03.039 32275214

[72] Giglioli IAC Pallavicini F Pedroli E Serino S Riva G. Augmented reality: a brand new challenge for the assessment and treatment of psychological disorders. Comput Math Methods Med.(2015) 2015:862942. 10.1155/2015/862942 26339283 PMC4538767

[73] Juan M-C Botella C Alcaniz M Banos RM Carrion C Melero M. An augmented reality system for treating psychological disorders: application to phobia to cockroaches. In: Third IEEE and ACM International Symposium on Mixed and Augmented Reality. Arlington, VA: IEEE (2004). p. 256–57.

[74] Juan M-C Alcaniz M Monserrat C Botella C Banos RM Guerrero B. Using augmented reality to treat phobias. IEEE Comput Graph Appl. (2005) 25:31–7. 10.1109/MCG.2005.143 16315475

[75] Botella C Juan MC Baños RM Alcañiz ML Guillén V Rey B. Mixing realities? An application of augmented reality for the treatment of cockroach phobia. CyberPsychol Behav. (2005) 8:162–71. 10.1089/cpb.2005.8.162 15938656

[76] Juan M-C Calatrava J. An augmented reality system for the treatment of phobia to small animals viewed via an optical see-through HMD: comparison with a similar system viewed via a video see-through HMD. Int J Hum Comput Interact. (2011) 27:436–49. 10.1080/10447318.2011.552059

[77] Juan M-C Joele D. A comparative study of the sense of presence and anxiety in an invisible marker versus a marker augmented reality system for the treatment of phobia towards small animals. Int J Hum Comput Stud. (2011) 69:440–53. 10.1016/j.ijhcs.2011.03.002

[78] Wrzesien M Burkhardt J-M Alcañiz M Botella C. How technology influences the therapeutic process: a comparative field evaluation of augmented reality and *in vivo* exposure therapy for phobia of small animals. In: Campos P, Graham N, Jorge J, Nunes N, Palanque P, Winckler M, editors. Human-Computer Interaction – INTERACT 2011. *Vol. 6946. Lecture Notes in Computer Science.* Berlin: Springer Berlin Heidelberg (2011). p. 523–40.

[79] Suso-Ribera C Fernández-Álvarez J García-Palacios A Hoffman HG Bretón-López J Baños RM. Virtual reality, augmented reality, and *in vivo* exposure therapy: a preliminary comparison of treatment efficacy in small animal phobia. Cyberpsychol Behav Soc Netw. (2019) 22:31–8. 10.1089/cyber.2017.0672 30335525 PMC6352498

[80] Botella C Pérez-Ara MÁ Bretón-López J Quero S García-Palacios A Baños RM. *In vivo* versus augmented reality exposure in the treatment of small animal phobia: a randomized controlled trial. PLoS ONE. (2016) 11:e0148237. 10.1371/journal.pone.0148237 26886423 PMC4757089

[81] Sahin NT Keshav NU Salisbury JP Vahabzadeh A. Safety and lack of negative effects of wearable augmented-reality social communication aid for children and adults with autism. J Clin Med. (2018) 7:188. 10.3390/jcm7080188 30061489 PMC6111791

[82] Bouchard S. Could virtual reality be effective in treating children with phobias? Expert Rev. Neurother. (2011) 11:207–13. 10.1586/ern.10.196 21306208

[83] Kim S Kim E. The use of virtual reality in psychiatry: a review. J Korean Acad Child Adolescent Psychiatry. (2020) 31:26–32. 10.5765/jkacap.190037 PMC7324842 32612410

[84] Garcia-Palacios A Botella C Hoffman H Fabregat S. Comparing acceptance and refusal rates of virtual reality exposure vs. *in vivo* exposure by patients with specific phobias. Cyber Psychol Behav. (2007) 10:722–4. 10.1089/cpb.2007.9962 17927544

[85] Garcia-Palacios A Hoffman H Carlin A Furness TA Botella C. Virtual reality in the treatment of spider phobia: a controlled study. Behav Res Ther. (2002) 40:983–93. 10.1016/S0005-7967(01)00068-7 12296495

[86] Minns S Levihn-Coon A Carl E Smits JAJ Miller W Howard D. Immersive 3D exposure-based treatment for spider fear: a randomized controlled trial. J. Anxiety Disord. 58:1–7. 10.1016/j.janxdis.2018.05.006 29909286

[87] Michaliszyn D Marchand A Bouchard S Martel M-O Poirier-Bisson J. A randomized, controlled clinical trial of *in virto* and *in vivo* exposure for spider phobia. Cyberpsychol Behav Soc Netw. (2010) 13:689–95. 10.1089/cyber.2009.0277 21142994

[88] Miloff A Lindner P Dafgård P Deak S Garke M Hamilton W. Automated virtual reality exposure therapy for spider phobia vs. *in-vivo* one-session treatment: a randomized non-inferiority trial. Behav Res Ther. (2019) 118:130–40. 10.1016/j.brat.2019.04.004 31075675

[89] Penate W Rivero F Vina C Herrero M Betancort M De la Fuente J. The equivalence between virtual and real feared stimuli in a phobic adult sample: a neuroimaging study. J Clin Med. (2019) 8:2139. 10.3390/jcm8122139 31817140 PMC6947488

[90] Deppermann S Notzon S Kroczek A Rosenbaum D Haeussinger FB Diemer J. Functional co-activation within the prefrontal cortex supports the maintenance of behavioural performance in fear-relevant situations before an ITBS modulated virtual reality challenge in participants with spider phobia. Behav Brain Res. (2016) 307:208–17. 10.1016/j.bbr.2016.03.028 26996315

[91] Carl E Stein AT Levihn-Coon A Pogue JR Rothbaum B Emmelkamp P. Virtual reality exposure therapy for anxiety and related disorders: a meta-analysis of randomized controlled trials. J Anxiety Disord. (2019) 61:27–36. 10.1016/j.janxdis.2018.08.003 30287083

[92] Powers MB Emmelkamp PMG. Virtual reality exposure therapy for anxiety disorders: a meta-analysis. J Anxiety Disord. (2008) 22:561–9. 10.1016/j.janxdis.2007.04.006 17544252

[93] Gregg L Tarrier N. Virtual reality in mental health. Soc Psychiatry Psychiatr Epidemiol. (2007) 42:343–54. 10.1007/s00127-007-0173-4 17431528

[94] Saredakis D Szpak A Birckhead B Keage HAD Rizzo A Loetscher T. Factors associated with virtual reality sickness in head-mounted displays: a systematic review and meta-analysis. Front Hum Neurosci. (2020) 14:96. 10.3389/fnhum.2020.00096 32300295 PMC7145389

[95] Straube T Glauer M Dilger S Mentzel H-J Miltner WHR. Effects of cognitive-behavioral therapy on brain activation in specific phobia. Neuroimage. (2006) 29:125–35. 10.1016/j.neuroimage.2005.07.007 16087353

